# Adsorption of Methylene Blue and Pb^2+^ by using acid-activated *Posidonia oceanica* waste

**DOI:** 10.1038/s41598-019-39945-1

**Published:** 2019-03-04

**Authors:** Randa R. Elmorsi, Shaimaa T. El-Wakeel, Waleed A. Shehab El-Dein, Hesham R. Lotfy, Wafaa E. Rashwan, Mohammed Nagah, Seham A. Shaaban, Sohair A. Sayed Ahmed, Iman Y. El-Sherif, Khaled S. Abou-El-Sherbini

**Affiliations:** 1 Marine Chemistry Lab., National Institute of Oceanography and Fisheries, Suez branch, Adabiyah-Suez road, Attaqa district, Suez, Egypt; 2 Department of Water Research, National Research Centre, 33 El Bohouth st. (former Eltahrir st.), P.O. 12622 Dokki, Giza Egypt; 3 Department of Mathematics & Engineering Physics, Faculty of Engineering, Mansoura University, El-Mansoura, Egypt; 4 Basic Sciences Department, Faculty of Engineering, Delta University for Science and Technology, Coastal High Way, Gamasa, Al-Dakahlia Egypt; 5 Department of Physical Chemistry, National Research Centre, 33 El Bohouth st. (former Eltahrir st.), P.O. 12622 Dokki, Giza Egypt; 6 Department of Microbial Chemistry, National Research Centre, 33 El Bohouth st. (former Eltahrir st.), P.O. 12622 Dokki, Giza Egypt; 7 Department of Catalysis, Petroleum Refining Division, Egyptian Petroleum Research Institute, Cairo, Egypt; 8 Department of Inorganic Chemistry, National Research Centre, 33 El Bohouth st. (former Eltahrir st.), P.O. 12622 Dokki, Giza Egypt

## Abstract

Dead leaves of seagrass *Posidonia oceanica* were activated by using one mol L^−1^ acetic acid and used as an eco-adsorbent for the removal of methylene blue (MB) and Pb^2+^ from aqueous solutions. The seagrass was characterized by chemical and physical measurements that confirmed the acid-activation of seagrass. The favourable conditions for MB and Pb^2+^ adsorption onto the activated seagrass (SG_a_) were determined to be a pH range of 2–12 and ≥6, an adsorbent dosage of 3.0 and 0.5 g L^−1^, respectively, and a shaking time of 30 min, which are suitable for a wide range of wastewaters. The equilibrium data were analysed using the Langmuir, Freundlich and Dubinin-Raduskavich-Kaganer (DRK) adsorption isotherm models. The Freundlich and DRK models best describe the adsorption processes of MB and Pb^2+^, on SG_a_ with capacities of 2681.9 and 631.13 mg g^−1^, respectively. The adsorption isotherm fitting and thermodynamic studies suggest that the adsorption mechanism of MB may combine electrostatic and physical multilayer adsorption processes, in which MB may be present as monomers as well as dimers and trimers which were confirmed from UV spectroscopy whereas Pb^2+^ is chemically adsorbed onto SG_a_. The pseudo-2^nd^-order kinetic model was utilized to investigate the kinetics of adsorption processes. The removal process was successfully applied for MB-spiked brackish waste water from Manzala Lake, Egypt, with removal efficiencies of 91.5–99.9%.

## Introduction

The discharge of toxic effluents containing heavy metals and dyes from a wide range of industries into water streams is of increasing concern due to the adverse effects of these effluents^1^. For example, in the aqueous dyeing textile industry, an average of 5.8 trillion litres of water are consumed per year, most of which are generated as wastewater containing 10–20% of the residual dye^2^. Heavy metal ions and many synthetic dyes are not biodegradable and cause severe health effects, even at low concentrations^3^. Hence, effective and economical methods for treating wastewater are necessary to reduce pollutant concentrations to acceptable levels.

Different techniques are used to remove heavy metal and dye pollutants from wastewater, such as precipitation, solvent extraction, membrane filtration, biodegradation and advanced oxidation^4,5^. These techniques lack the advantages of being fast, economical and/or eco-friendly. However, adsorption has proven to be effective and serves as an alternative treatment technique for the removal of hazards from wastewaters^6,7^, although inexpensive adsorbents capable of maintaining their efficiency especially in the presence of a saline background are rare^8,9^.

Although adsorption by activated carbon has long been utilized to remove pollutants from wastewater, its high production cost and regeneration difficulties have limited its use^10,11^. and thus, attention has shifted to the identification of cheaper and more efficient adsorbent alternatives, such as abundantly available natural agricultural wastes^12,13^.

The adsorption of various pollutants on bio-waste provides a method for evaluating the utilization of waste biomasses. In addition, this process prevents environmental pollution by utilizing these biomasses and decreasing the cost of wastewater treatment^14,15^.

Methylene blue (MB) is frequently utilized as a model cationic dye in adsorption studies^16–18^. Also, lead (Pb^2+^) is an environmental risk factor that represented approximately 0.6% of the global disease burden found in 2010^19^. The ability of several low-cost agricultural waste materials to remove MB and Pb^2+^ from effluents has been studied. Some of these new adsorbents include, banana waste^13,20^, oiltea shell^21^, algae^17,22^, and marine seaweed^18^. Algae have also been identified as potential Pb^2+^ biosorbents due to their high content of binding sites, such as carboxyl, sulfonate, amine and hydroxyl groups^22,23^.

On the other hand, *Posidonia oceanica* is an endemic dominant seagrass (SG), covering approximately 50,000 km^2^ of the coastal sandy areas in the Mediterranean Sea and is involved in seawater oxygenation, fauna protection and littoral erosion prevention^24,25^. *P*. *oceanica* is considered a pollution-biomonitor as it naturally reclaims heavy metal ions from seawater in its temporal organs, such as its leaves, hosting varying amounts of heavy metal ions, from a few mg kg^−1^ of As, Cd and Pb up to several tens of mg kg^−1^ of Zn^26–28^. The ball-shaped dead leaves of this seagrass form large wastes along the Mediterranean coast, thereby imposing an environmental threat^25,29^. These dead leaves are a natural cation-imprinted lignocellulosic framework that is adopted to highly saline environments^29^. Hence they were utilized (either directly or after modification) as an bioadsorbent for MB^30,31^ and Pb^2+^ ions^32,33^ from wastewater. These procedures are tedious and/or yield low adsorption capacities. The current study presents a simple and new acid-activation procedure for the recycling of SG eco-waste as an green adsorbent for cationic pollutants from brackish water.

## Material and Methods

### Material

Glacial acetic acid and a standard 1000 mg L^−1^ of Pb^2+^ as Pb(NO_3_)_2_ were purchased from Doummar & Sons Co. (Adra, Syria) and Merck (USA), respectively. MB chloride hydrate (3,7-bis(dimethylamino)-phenothiazin-5-ium chloride), which has a formula of C_16_H_18_ClN_3_S·xH_2_O and a dye content >96.0%, was purchased from S D Fine-Chem Limited (Mumbai, India). Stock solution of MB (1000 mg L^−1^) was prepared by dissolving 1 g in one litre of distilled water. Other chemicals and reagents were of grade Puriss from Sigma–Aldrich unless otherwise stated.

SG waste (S1) was collected from the shore of Marsa Matrouh, Egypt, washed thoroughly in tap water, skimmed, and dried in air. To examine the practical application, surface water samples were collected from 12 sampling sites along Lake Manzala, Egypt, as recently detailed^34^.

### Equipments

The concentrations of Al, As, B, Ca, Cd, Cr, Cu, Fe, Mg and Zn were determined by inductively coupled plasma-optical emission spectrometry (ICP-OES) instrument 5100 (Agilent, USA). A 0.1 g portion of the plant sample was digested in aqua regia, and the analysis of metal contents was conducted according to standard methods^35^. Calibration curves were performed using diluted solutions prepared from 1000 mg L^−1^ of element standard solutions (Merck USA). All tests were carried out in triplicate.

An automatic elemental Vario EL cube instrument (Elementar, Germany) was used to determine the percentage of C, H, N and S in the samples. The pH-metric titration measurement was performed using an automatic potentiometer 848 Titrino plus (Metrohm, Switzerland). Exactly 100 mg of the investigated sample was added to 25 mL of 0.5 mol L^−1^ KCl and titrated against 0.0073 mol L^−1^ KOH+ 0.5 mol L^−1^ KCl at 25 °C at a rate of 1.0 mL min^−1^. The point of zero charge (pH_PZC_) of the adsorbents was determined as detailed in S2. Nitrogen adsorption/desorption isotherms were performed to determine the surface texture parameters at liquid nitrogen temperature (−196 °C) using an automatic gas sorption apparatus Nova 3200S (Quantachrome, USA). Prior to such measurements, samples were perfectly degassed at 100 °C for four hours under vacuum pressure 5 × 10^−4^ Pa. The thermal gravimetric analysis (TGA) was conducted on a thermal analyzer Q600 SDT Quickstart (TA Instruments, USA) in N_2_. Fourier transform infrared (FTIR) spectra were recorded on a Nicolet iS10 instrument (Thermo-Fisher Scientific, USA) in KBr pellets. The pH of each sample solution was adjusted with NaOH and HNO_3_ solutions using a Chemcadet 5986–50 pH/ion/mV meter (Cole-Parmer, USA).

Electronic spectra of MB solutions were recorded on a UV/VIS spectrophotometer 2100 (Unico, USA) at 663.8 nm. The concentration of MB was determined by using a calibration curve made from standard MB solutions.

### Methodology

#### Chemical composition of raw material

The analysis of the organic composition is described in S3.

#### Treatment of SG

SG was washed thoroughly with distilled water, dried at 80 °C to a constant mass, sieved to remove sand, pulverized in a blinder, and sieved through a 1.0 mm screen. The obtained material is denoted SG_p_ from which approximately 200 g was soaked in one litre of one mol L^−1^ acetic acid overnight, skimmed, washed thoroughly with distilled water, dried at 80 °C for 24 h, and stored in polyethylene vials. This treated material is denoted ‘SG_a_’.

#### Adsorption studies

The adsorption experiments were carried out at room temperature (23 ± 1.0 °C) and the natural initial pH values of MB and Pb^2+^ solutions; 5.8 and 5.3, respectively, unless otherwise stated.

For the MB adsorption isotherm studies, batch experiments were conducted using 5 g L^−1^ of the adsorbent suspensions with initial concentrations ranging from 2 to 500 mg L^−1^ under shaking for 10–90 min. Similar experiments were done using a 20 mg L^−1^ Pb^2+^ solution and 0.5 g L^−1^ of adsorbent.

The amounts of adsorbed MB and Pb^2+^ (mg g^−1^) at equilibrium (*q*_*e*_) and at time *t* (q_t_) were calculated from the mass balance expressions given by the following equations:

1 $${q}_{e}=\frac{({C}_{0}-{C}_{e})}{{\rm{m}}}V$$

2 $${q}_{t}=\frac{({C}_{0}-{C}_{t})}{{\rm{m}}}V$$

where *C*_0_, *C*_*e*_, and *C*_*t*_ are the liquid-phase concentrations (mg L^−1^) of MB or Pb^2+^ at the start, at equilibrium and at time *t*, respectively. *V*(L) is the volume of the solution and *m*(g) is the mass of adsorbent.

The effect of the SG_a_ dosage on the adsorption of each of MB and Pb^2+^ was studied at an initial concentration of 40 mg L^−1^ MB and 20 mg L^−1^ Pb^2+^, with a shaking time of 60 min. The suspensions were then centrifuged, and the residual concentration of adsorbate in the supernatant solution was determined.

The thermodynamic studies of the adsorption of MB on SG_a_ were performed at 23, 30 and 37 °C by adding 2.5 g L^−1^ of the adsorbent to a solution with an initial MB concentrations of 20, 120 and 172 mg L^−1^ and shaking for 60 min.

To investigate the effect of the initial pH on the adsorption behaviour, 40 or 20 mg L^−1^ of MB or Pb^2+^ was chosen as the initial concentration and a SG_a_ dosage of 5 or 0.5 g L^−1^, respectively was used. The initial pH (2.0–11.4) of the MB or (2.0–6.0) Pb^2+^ solution was controlled using 0.1 M NaOH and HCl or HNO_3_ solutions. The resulting suspensions were shaken for 60 min, centrifuged, and the residual concentrations of adsorbates in solution were determined.

The loaded adsorbents, prepared by adding 100 mg of SG_a_ to 20 mL of 2 or 100 mg L^−1^ MB at an initial pH of 5.8 and shaking for 60 min, were washed several times with distilled water, dried at 80 °C for 24 h and stored for analysis.

#### Application

A batch experiment was performed for the adsorption of MB from Lake Manzala water samples spiked with 40 mg L^−1^ MB. A dosage of 5 g L^−1^ was stirred in 20 mL of the water sample for 60 min at ambient pH and room temperature. Then, the samples were filtered, and both the initial (*C*_0_) and residual (*C*_*r*_) MB concentrations were determined. The removal efficiency (*E*_*R*_%) was determined from the following formula:

3 $${E}_{R}( \% )=\frac{{C}_{0}-{C}_{r}}{{C}_{0}}\times 100$$

## Results and Discussion

### Characterization of SG_p_ and SG_a_

A comparison of the chemical analysis of SG_p_ and SG_a_ with reported results (Table 1) indicated the presence of typical components. The cellulose, hemicellulose, and lignin contents of SG_p_ and its activated product SG_a_ were mostly comparable or close to the reported data^36,37^. However, the ash content of SGa was remarkably reduced to approximately one third of its original value in SG_p_ which may be attributed to the leaching out of some bonded metal ions. This was confirmed by the analysis of some common and trace metal ions in SG_p_ and SG_a_. Despite the metal contents in both samples was comparable to reported values but SG_a_ possessed obviously lower content of most of the analysed elements and their summation compared with the untreated plant leaves.

**Table 1:** Chemical analysis of SG_p_ and SG_a_ compared with reported values of *P*. *oceanica*.

Chemical composition (%)						
Component	This work		^36^		^37^	
	(SG)	(SG_a_)				
Cellulose	35.12	35.00	40		—	
Hemicellulose	22.52	25.03	21.8		—	
Lignin	18.73	17.82	9.1		—	
Ash	12.03	3.74	12.0		13.3–30.6	
Moisture	2.60	2.35	—		—	
Total solids	97.40	97.65	—		—	
**Some metal and metalloid contents (mg kg** ^**−1**^ **)**						
**Element**	**This work**		^37^	^28^	^38^	^39^
	**(SG**_**p**_ **)**	**(SG**_**a**_ **)**				
Al	300	320	—	—	—	—
As	2.5	2	—	2.73	—	—
B	2000	65	1013–4055	—	—	—
Ca	10850	10750	562–56289	—	—	—
Cd	2.5	2	0.5–1.1	1.34	0.23–0.88	2.1–5.38
Cr	47.5	12	3.2–18.8	2.47	—	0.20–1.27
Cu	10	2	17.8–55.5	11.6	0.37–24.9	—
Fe	875	780	2093–7455	—	93.6–1400	—
Mg	4800	600	7667–17125	—	—	—
Zn	10	2	53.2–69.5	115	10.6–63.2	1.4–1.8
Summation	18897.5	12535				
**C, H, N and S contents (%)**						
**Element**	**This work**		^37^		^40^	
	**SG**_**p**_	**SG**_**a**_				
C	37.86	38.38	35. < 5–47.7		35.5	
N	0.42	0.26	0.29–1.02		0.16	
H	4.93	5.06	—		3.6	
S	0.16	0.16	—		0.18	

**Figure 1:**
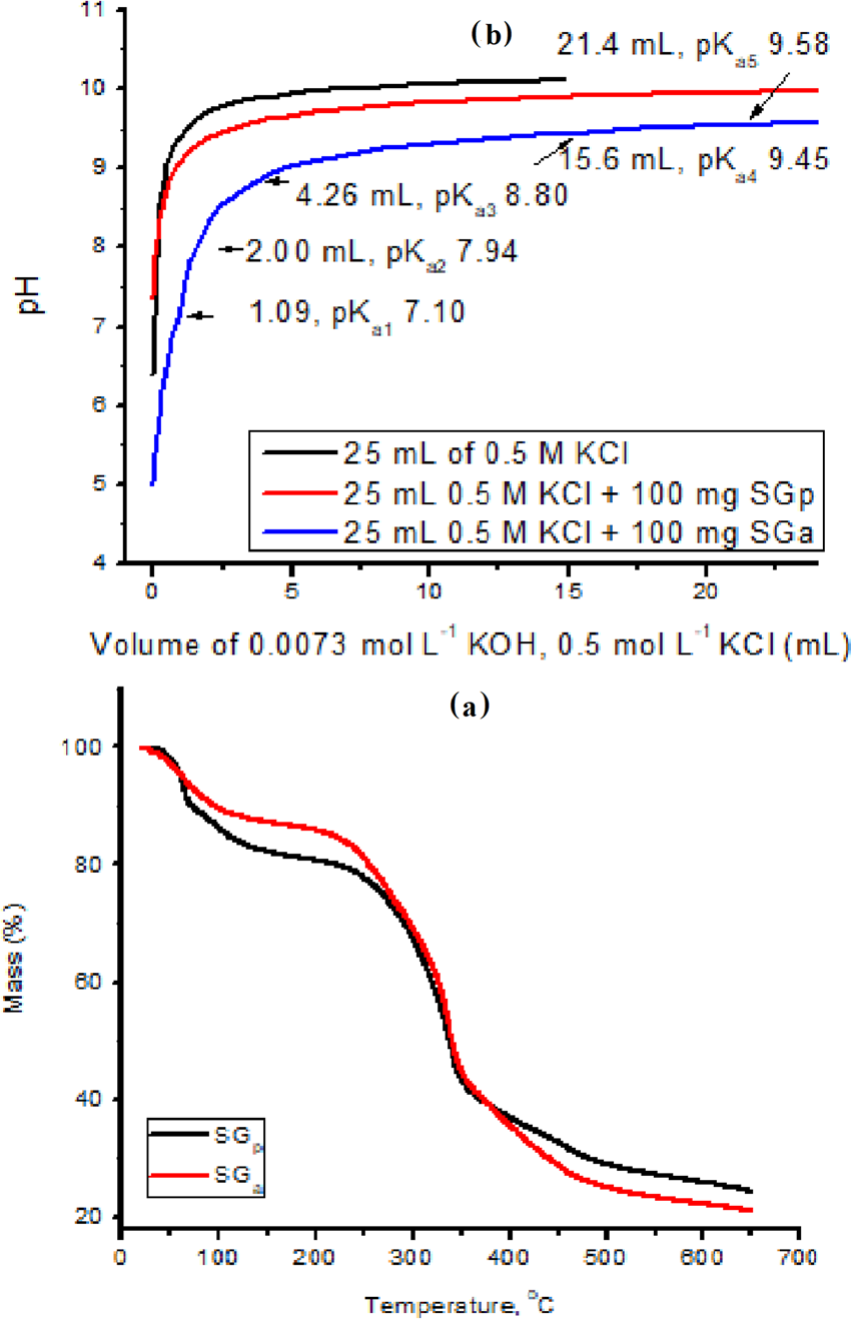
TGA (**a**) and pH-metric titration versus 0.0073 mol L^−1^ KOH (**b**) of SG before and after acid treatment of SG_p_ and SG_a_.

The observed slight increase in the C and H contents of SG_a_ relative to those of SG_p_ is in accordance with the obvious increase in hemicellulose and lignin that may be also attributed to the removal of inorganic deposits. This was further confirmed from TGA of SG_a_ and SG_p_ (Fig. 1a) where a 9.14% larger mass loss in the temperature range 200–500 °C, attributed to the loss of organic content^41^, was observed for SG_a_ compared with SG_p_. This mass loss stage is formed of two distinct stages; a fast one from 230 to 350 °C and a slow one from 350 to 500 °C which may be due to the thermal degradation of cellulose-hemicellulose and lignin, respectively. On the other hand, the activated seagrass shows a lesser residues at 650 °C, which is an important factor if it is used as a bio-fuel^42,43^. Moreover, SG_a_ contains lower amounts of nitrogen and sulphur than SG_p_ and other reported SG samples^37,40^.

The pH-metric titration of SG_a_ versus 0.0073 mol L^−1^ KOH and 0.5 mol L^−1^ KCl (Fig. 1b) suggested a remarkable enhancement in the acidity relative to that of SG_p_, which may be attributed to a partial hydrolysis of the ester groups, a release of the carboxylic groups engaged in ionic bonding and a decomposition of the probable basic components, such as carbonates. This enhanced acidity was indicated by an increase in the consumed volume of basic titrant by SG_a_ (3.71 mL, 0.271 mmol g^−1^) compared with that consumed by SG_p_ at pH 9.03 and by the obvious shift in the pH_pzc_ of SG_a_ to the more acidic value of 6.6 compared with the value of 7.4 obtained for SG_p_ (S4). Additionally, in the case of SG_a_, the titration curve was more complicated than that of SG_p_, showing almost five stages at basic titrant volumes of 1.09, 2.00, 4.26, 15.6 and 21.4 mL, corresponding to pKa values of 7.10, 7.94, 8.80, 9.45 and 9.58, respectively and indicating the presence of at least five exchangeable protons with diverse acidities.

The nitrogen adsorption/desorption isotherms of SG_p_ and SG_a_ (S5) correspond to type III with a hysteresis loop according to IUPAC classification typically obtained in case of microporous materials indicating unrestricted multilayer formation process. S_BET_ values of SG_p_ and SG_a_ were 67.0 and 146.7 m^2^ g^−1^, and average pore diameters were 1.255 and 0.738 nm, respectively. The increase in S_BET_ for SG_a_ may be related to channel cleansing, and to elimination of aggregated impurities that is in accordance with the chemical analysis (Table 1), TGA and pH-metric titrations (Fig. 1). The textural properties of the two adsorbents are summarized in Table 2:

**Table 2:** Textural properties of SG_p_ and SG_a_.

Sample	BET (m^2^ g^−1^)	Average pore diameter (nm)	Pore volume (cm^3^ g^−1^)	Micropore area (m^2^ g^−1^)
SG_p_	67.0	1.26	0.021	51.48
SG_a_	146.7	0.74	0.0271	127.59

**Figure 2:**
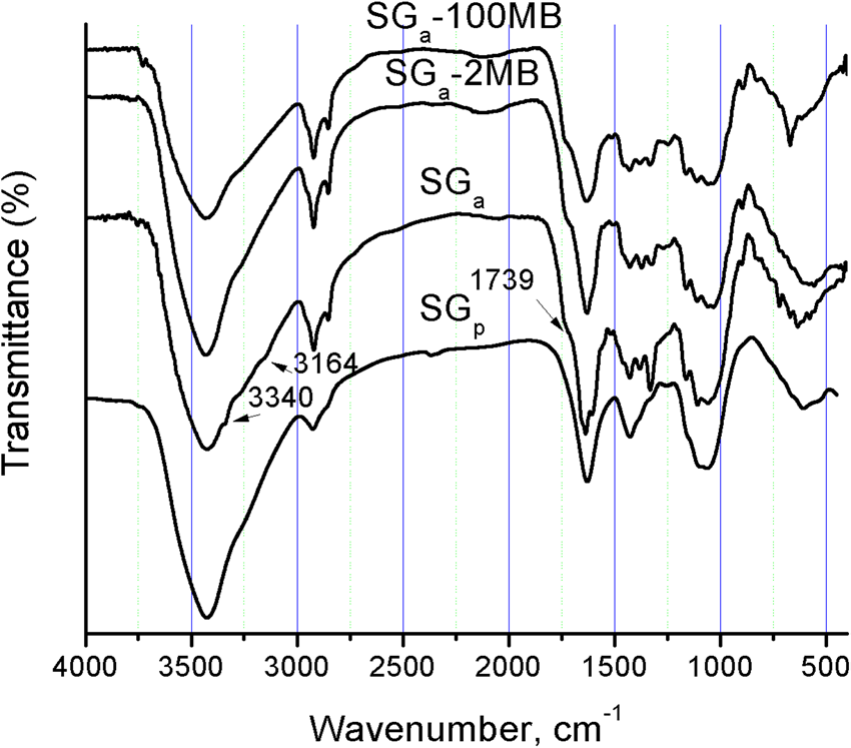
FTIR absorption spectra of SG_a_, SG_p_, SG_a_-2 MB and SG_a_-100 MB.

The FTIR spectra of SG_a_, SG_p_ and loaded SG_a_-2 MB or −100 MB (seagrass loaded with 0.39 or 18.66 mg g^−1^ of MB, respectively) are presented in Fig. 2. A typical IR spectrum of the seagrass *P*. *oceanica* was observed for SG_p_^29,37^. The bands at 3426 (broad), 2925, 2857, 1629(wide), 1428, 1100 and 1061 cm^−1^ are assignable to hydrogen-bonded ν_OH_; asymmetric ν_aliphatic CH_, symmetric ν_aliphatic CH_, the overlapped vibrations from δ_OH_, aromatic C=C and asymmetric COO^−^ groups; overlapped symmetric COO^−^ and δ_CH2_ vibrations; C-O-C pyranose, and C-O asymmetric bridge stretching vibrations, respectively^44^. The spectrum of SG_a_ was more resolved and exhibited more detailed fine peaks, especially in the IR absorption region of 400–1850 cm^−1^. New bands were observed at 3340, 3164, 1739, 1658, 1599, and 1382 cm^−1^, which are assignable to two different ν_OH_ vibrations, free carboxylic C=O, pectin, lignin and OH deformations, respectively. The observation of a vibration for free COOH groups supports the possible release of the acidic sites bound to cationic species and/or the occurrence of a partial degradation. This is in accordance with the above-discussed measurements and the reported effect of acetic acid on plant fibres and cellulose^45^.

The spectra of the MB-loaded SG_a_ showed a blue shift in the wave number from 3426 cm^−1^ to 3433–3435 cm^−1^ and a red shift from 1636 cm^−1^ to 1629–1631 cm^−1^ which may indicate that the OH groups are involved in the adsorption of MB. This was also supported by the disappearance of the shoulders at 3340, 3164, 1658 and 1543 cm^−1^.

### Adsorption studies

#### Effect of initial solution pH

**Figure 3:**
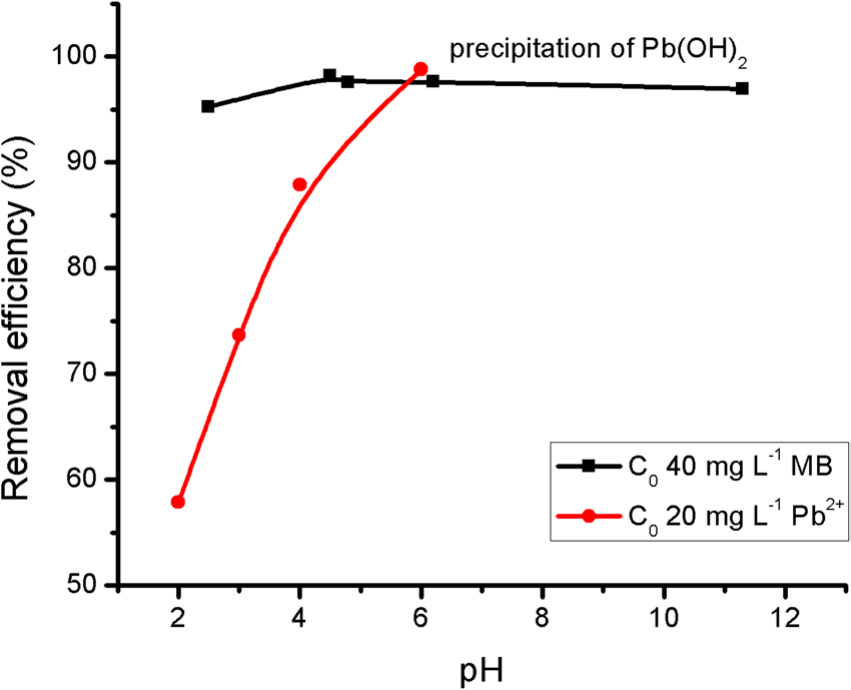
Effect of solution pH on the percent removal efficiency of MB or Pb^2+^ onto SG_a_.

The effect of the initial solution pH on *E*_*R*_% of MB and Pb^2+^ on SG_a_ is represented in Fig. 3. The removal efficiency of MB was high (>95%) and nearly independent of the pH within the examined pH range. This may be due to the strong MB/SG_a_ hydrophobic interactions, which is a general tendency of organic molecules to associate physically with the adsorbents. In the case of Pb^2+^, *E*_*R*_% increased with increasing pH until reaching a maximum removal efficiency at pH 6 while higher pH range was not studied to avoid the precipitation of Pb(OH)_2_. The observed difference in behaviour for the investigated adsorbates supports the adoption of different adsorption mechanisms originating from their organic and inorganic natures. In the case of Pb^2+^, at low pH, the relatively higher concentration of H^+^ ions may effectively compete with the metal cations for the adsorption sites and hinder their adsorption. In addition, the positive charge of SG_a_ at low pH may repel the Pb^2+^ ions. However, as the pH increases, the H^+^ ion concentration in solution and the positive charge of SG_a_ decreases, as indicated by its pH_pzc_ value, which provides more opportunities for adsorption.

#### Effect of contact time

**Figure 4:**
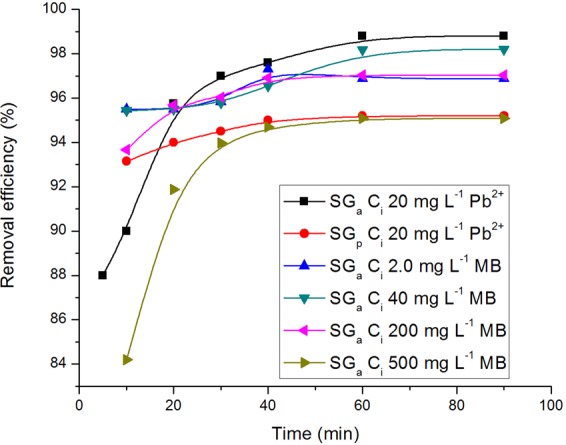
The effect of shaking time on the removal efficiency (%) of MB or Pb^2+^ applying initial pH values 5.8 and 5.3 and adsorbent dosage of 5 and 0.5 g L^−1^, respectively, on the adsorbents SG_a_ or SG_p_.

The effect of the contact time on the adsorption of MB and Pb^2+^ by SG_a_ and SG_p_ is shown in Fig. 4. Equilibria were nearly reached within 30 min, after which the removal efficiency slightly increased. Pb^2+^ is adsorbed onto SG_a_ more favourably than onto SG_p_, which is in accordance with the suggested enhancement of the acidity. Accordingly, 30 min was enough for attaining removal efficiencies more than 93% of the initial concentration that were close to the maximum values obtained at equilibration time 60 min for MB and Pb^2+^ on SG_a_ and SG_p_.

**Figure 5:**
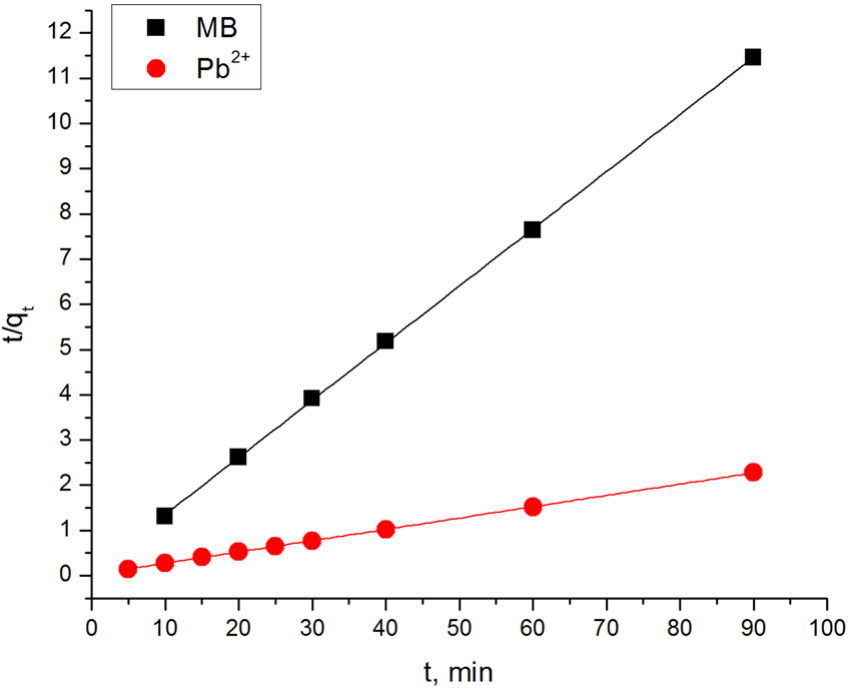
Plot of t/q_t_ versus t for the application of pseudo 2^nd^ order kinetic model. *C*_*i*_ of MB and Pb^2+^ are 200 and 20 mg L^−1^, SG_a_ adsorbent dosage of 5 and 0.5 g L^−1^, and initial pH values 5.8 and 5.3, respectively.

The experimental data obtained from stirring time/adsorption correlations (Fig. 4) for the initial concentrations 200 and 20 mg L^−1^ of MB and Pb^2+^, respectively, using SG_a_, were applied to the pseudo-1^st^- order and pseudo-2^nd^-order adsorption models (S6). The fitting results (Table 3) show that the pseudo-2^nd^-order model fits the adsorption processes of MB and Pb^2+^ onto SG_a_ well, regardless of their initial concentration, as illustrated by the good agreement between the experimental and calculated equilibrium adsorption capacities, *q*_*e*_, and the high values of R^2^ ≥ 0.99 (Fig. 5). This behaviour is independent of the experimental variables, i.e., the adsorbent, initial concentration and pH^46^. The obtained results suggest that a heterogeneous adsorption mechanism is likely to be responsible for the uptake of MB and Pb^2+^ ions.

**Table 3:** Kinetic parameters for the adsorption of MB and Pb^2+^ onto SG_a_.

Pseudo-first order				
*Adsorbate* (*C*_0_ *mgL*^−1^)	*q*_e_ (mg g^−1^)		*K*_1_(min^−1^)	*R* ^2^
	(calculated)	(experiment)		
MB (2.0)	0.007	0.388	0.01	0.750
MB (40)	0.24	7.85	0.01	0.890
MB (200)	2.22	38.82	0.06	0.920
MB (500)	33.43	95.08	0.11	0.998
Pb^2+^ (20)	6.86	39.52	0.07	0.982
**Pseudo-second order**				
***Adsorbate*** **(** ***C***_***0***_ ***mgL*** ^**−*1***^ **)**	***q***_**e**_ **(mg g** ^**−1**^ **)**		**K**_**2**_ **(g g** ^**−1**^ **min** ^**−1**^ **)**	***R*** ^**2**^
	**(calculated)**	**(experiment)**		
MB (2.0)	0.389	0.388	10.807	1.000
MB (40)	7.91	7.85	0.185	1.000
MB (200)	39.06	38.82	0.068	1.000
MB (500)	96.15	95.08	0.010	0.999
Pb^2+^	40.16	39.52	0.022	1.000

#### Effect of adsorbent dose

**Figure 6:**
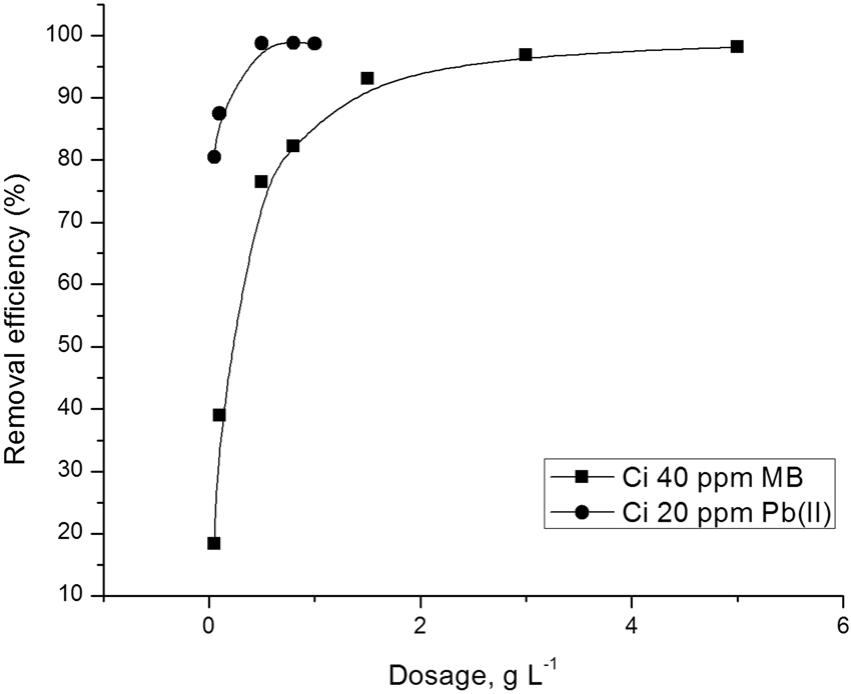
Effect of dosage on the removal efficiency of MB and Pb^2+^ by SG_a_.

The effect of the adsorbent dose on the adsorption of MB and Pb^2+^ on SG_a_ is shown in Fig. 6. The removal percentage increased with increasing adsorbent dose and exceeded 95% with an adsorbent dose of 3 and 0.5 g L^−1^ in the case of MB and Pb^2+^, respectively. These results could be due to the fact that more adsorption sites become available with increasing adsorbent dose, allowing more adsorbate ions to adhere to it^47^. The apparent difference in the optimum dosage for MB and Pb^2+^ may be explained by the large size difference between both adsorbates.

#### Effect of initial concentration

**Figure 7:**
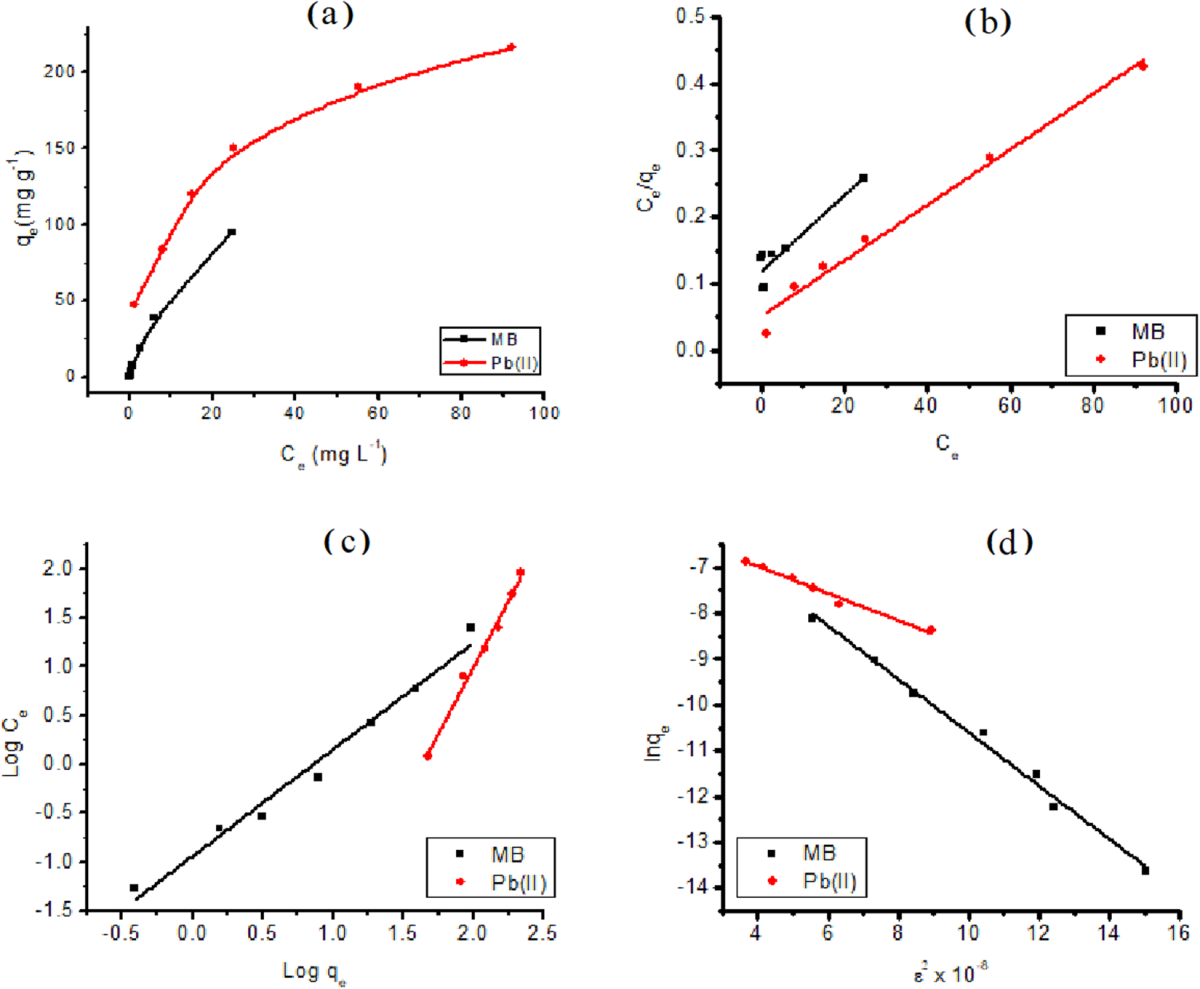
Adsorption isotherms of MB or Pb^2+^ onto SG_a_ (**a**) and their linear fitting to Langmuir (**b**), Freundlich (**c**) and DRK (d) adsorption models. *ε* (adsorption potential) = $RT\,ln(\frac{1}{1+{C}_{e}})$.

The adsorption isotherms of MB dye and Pb ions on SG_a_ are shown in Fig. 7 which are characteristic of types L1 and H2, respectively, according to the classification proposed by Giles *et al*.^48^ for liquid-solid interactions. They correspond to moderate and high interactions, respectively, between the solid and the adsorbate, which is typical of microporous solids.

The adsorption isotherms were examined with three adsorption isotherm models, namely, the Langmuir, Freundlich and Dubinin–Radushkevich–Kaganer (DRK) models (Fig. 7) according to (S7). Table 4 summarizes the parameters of the isotherm models. The R^2^ values indicate the good fitness of the studied adsorption models for MB and Pb^2+^ on SG_a_ except for the Langmuir isotherm model for MB. However, the best model that describes the adsorption of MB and Pb^2+^ on SG_a_ is that of DRK and Freundlich, respectively. The monolayer coverage of Pb^2+^ on the adsorbent was 238 mg g^−1^. The R_L_ values were found to be less than 1 and greater than zero (0 < R_L_ < 1), indicating that the adsorption processes are favourable. The values of 1/n indicate favourable adsorption of MB and Pb^2+^ onto SG_a_ within the studied ion concentrations.

**Table 4:** Isotherm model parameters for the adsorption of MB and Pb^2+^ onto SG_a_.

Langmuir isotherm parameters			
Adsorbate	q_max_ (mg g^−1^)	*R*_*L*_	*R* ^2^
MB	175.44	0.040–0.912	0.856
Pb^2+^	238.09	0.057–0.327	0.986
**Freundlich isotherm parameters**			
**Adsorbate**	**l/** ***n***	***K***_**F**_ **(mg/g)**	***R*** ^**2**^
MB	0.897	7.218	0.980
Pb^2+^	0.36	43.66	0.989
**DRK isotherm parameters**			
**Adsorbate**	***q***_**max**_ **(mg g** ^**−1**^ **)**	**E (kJ/mol)**	***R*** ^***2***^
MB	2681.9	9.266	0.992
Pb^2+^	631.13	12.99	0.984

**Figure 8:**
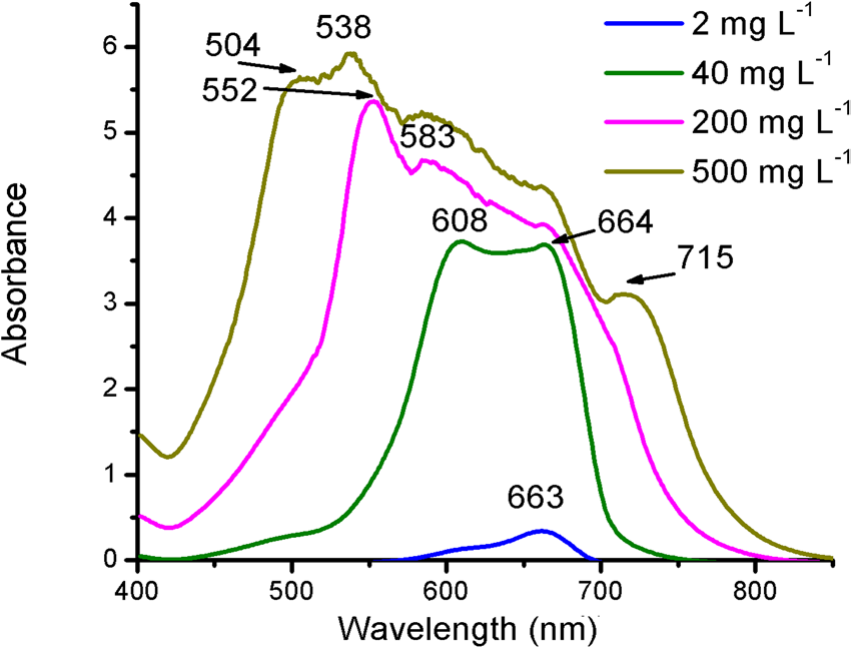
Effect of MB concentration on its UV absorption spectra in aqueous solutions.

The mean free energy, derived from DRK model, provides information about whether the mechanism of adsorption is physical or chemical. Accordingly, the positive adsorption energies of E = 9.27 and 13.0 kJ mol^−1^ obtained for MB and Pb^2+^, respectively, indicate that these adsorbates are chemically adsorbed onto SG_a_ in endothermic processes. Considering the large difference in the size of the adsorbates, where MB ≫ Pb^2+^, the q_max_ value for MB was expected to be substantially smaller than that for Pb^2+^ based on the limited surface area available on SG_a_. In contrast, the DRK model gave q_max_ values of 2681.9 and 631.13 mg g^−1^ for MB and Pb^2+^, respectively, thereby suggesting that the adsorption mechanism of MB may combine electrostatic and physical multilayer adsorption processes. The molar ratio of q_max_ values for MB and Pb^2+^ is 2.75:1, which indicates that the adsorbed MB species may be present as dimers and trimers if the same adsorption sites of SG_a_ host both adsorbates^49,50^. This was expected based on the strong tendency of MB to polymerize in H and J types in aqueous solutions at concentration higher than 20 μmol L^−1^ ^49–53^. To visualize the presence of MB aggregates in solutions at the studied MB concentration range 2.0–500 mg L^−1^, UV absorption spectra was measured for these solutions and shown in Fig. 8. Obviously, the monomer phase (λ_max_ = 663–664 nm)^50^ prevails at concentration of MB of 2.0–40 mg L^−1^, whereas the dimer and trimer species prevail with increasing concentration as convinced from the observation of their λ_max_ at 608 and 583 nm, which is close to the reported values 605^50^ and 580 nm^54^, respectively.

#### Effect of temperature

An important factor that strongly affects the adsorption process is the temperature, and its influence was studied for MB. The thermodynamic parameters of MB adsorption onto SG_a_ were calculated to estimate the changes in the free energy (∆G_ads_), enthalpy (∆H_ads_) and entropy (∆S_ads_) of the adsorption process. As the adsorption isotherm could not be fitted to the Langmuir model, and assuming that C_e_ = a_e_ at C_e_ → 0, then the distribution coefficient (K_d_, mL g^−1^) was obtained at C_e_ → 0 from the extrapolation of the linear plot of K_d_ vs. C_e_, where K_d_ equals q_e_/C_e_. For calculations, the following relationships were employed^55^:

4 $${\rm{\Delta }}{G}_{ads}=-\,RTln{K}_{d}$$

∆H_ads_ and ∆S_ads_ were calculated from Van’t Hoff’s equation.

5 $${\rm{\Delta }}{G}_{ads}={\rm{\Delta }}{H}_{ads}-T{\rm{\Delta }}{S}_{ads}$$

**Figure 9:**
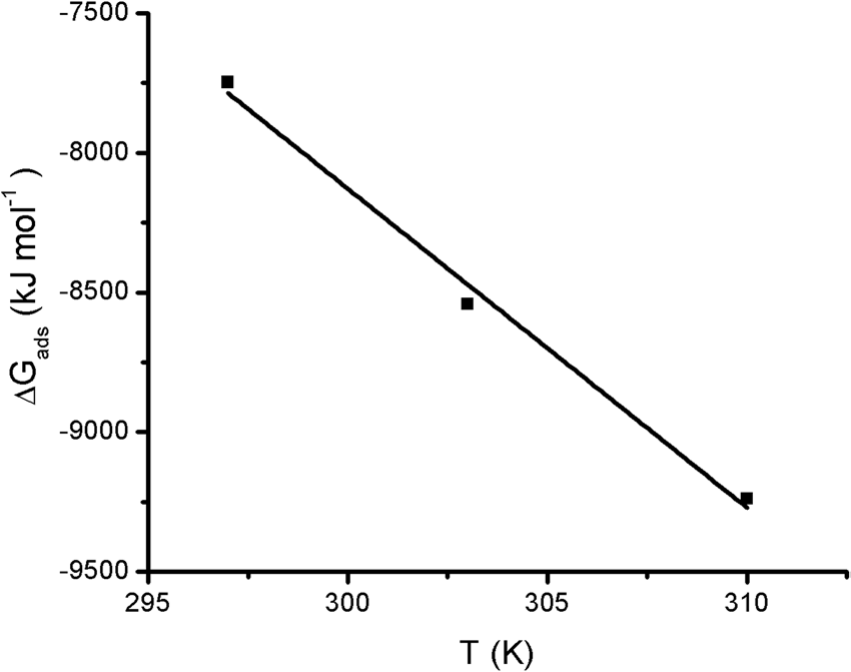
Effect of temperature on ∆G_ads_ of the adsorption of MB on SG_a_.

The slope and intercept of the plot of ∆G_ads_ vs. T (Fig. 9) derived from Eq. (5) were used to calculate ∆H_ads_ and ∆S_ads_. ∆G_ads_ were negative −7.748, −8.542 and −9.239 kJ mol^−1^, at T 297, 303 and 310, respectively, suggesting the spontaneous nature of the adsorption process and meanwhile confirming the feasibility of the adsorption process. ∆H_ads_ was 26.156 kJ mol^−1^, which indicates that the adsorption of MB on SG_a_ occurs through both endothermic physical (possibly dimerization and trimerization in solution) and chemical processes^56^. A small positive value was obtained for ∆S_ads_ (0.114 kJ K^−1^ mol^−1^), indicating a slight increase in the system randomness. The increase in ∆S is unexpected in the adsorption processes considering the loss of one degree of freedom due to the transport of freely moving dissolved MB^+^ ions from aqueous solution to the adsorbent phase. However, this was reported for many organic adsorbate/adsorbent systems such as MB/Bacillus subtilis^57^, MB/wheat shells^58^, 2-nitroanilin/active carbon^59^, and MB/citrus limetta peel^13^. This was explained by the bulk and the organic nature of the dye and also by a possible structural deformation occurring during the sorption process^59^. Another reason is the possible formation of many conformations of MB species (present in aqueous solution as J and H types of monomer, dimer and trimer species)^49–54^ on the adsorbent surface during the multilayer-physical adsorption.

**Figure 10:**
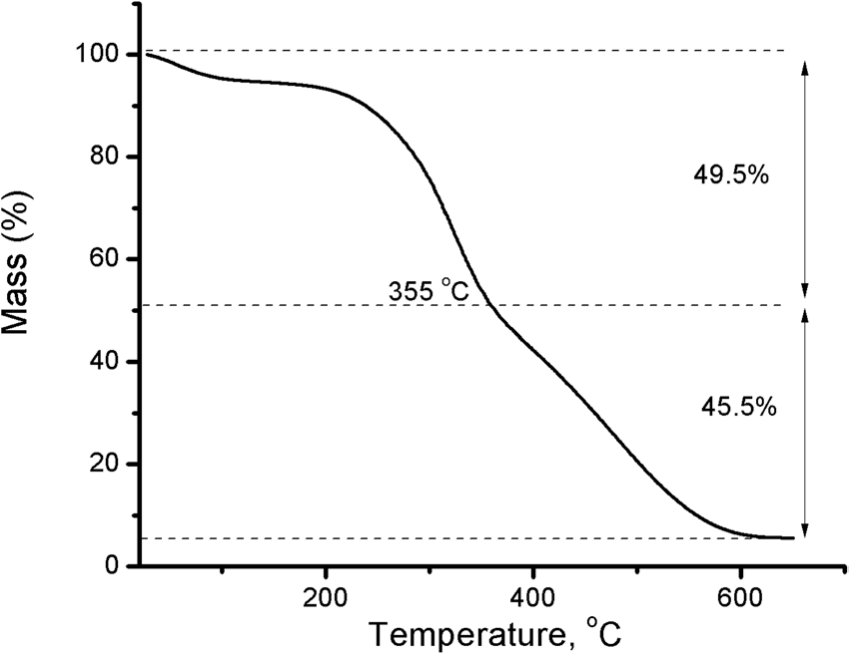
Thermal analysis of SG_a_ loaded with MB (6.44 mEq g^−1^).

The maximum adsorption capacity of SG_a_ was explored to verify the theoretical capacity suggested by DRK model and the observable dependence on temperature. One g of SG_a_ was loaded with MB using *C*_0_ 2.0 and 20 g L^−1^ (100 mL of each) in two subsequent batch experiments for 60 min at 60 °C. The obtained adsorption capacities were 96.5 and 93.3%, respectively, and the total loading capacity was 6.44 mEq g^−1^ which was close to the theoretical value that obtained from DRK model. The loaded sample MB-SG_a_ of this experiment was subjected to TGA analysis to assure the loading of MB as shown in Fig. 10. Obviously, the mass loss percentage of the organic content increased to approximately 90% compared with that of SG_a_ 67% (Fig. 1). The second organic content loss stage suggested in section 3.1 to be due to lignin is responsible for this mass loss increase that may be attributed to MB pyrolusis as both compounds are aromatic in nature.

The low nitrogen and sulphur contents of SG_a_ (Table 1) are advantageous key parameters compared with other biomasses^43^ that permit the safe NO_x_ and SO_x_ emissions if the spent adsorbent (dye-loaded SG_a_) may be utilized as a safe bio-fuel.

Accordingly, the optimum conditions for MB and Pb^2+^ adsorption onto SG_a_ are a pH ranges of 2–12 and ≥6, and a dosage of ≥3 and 0.5 g L^−1^, respectively, at shaking time of ≥30 min, which are suitable for a wide range of wastewaters. The adsorption parameters obtained for SG_a_ in comparison with those reported earlier for adsorption of MB or Pb^2+^ ions onto various adsorbents including modified and unmodified SG (S8) revealed that SG_a_ is a promising eco-waste adsorbent for removing MB or Pb^2+^ ions within proper equilibration time and superior adsorption capacity.

### Application

The removal efficiencies of MB by SG_a_ from Lake Manzala water samples were examined after spiking with 40 mg L^−1^ MB. *E*_*R*_% of the spiked MB ranged from 91.5 to 99.9%, as shown in (S9). These values indicate the feasibility of SG_a_ as an effective and powerful adsorbent for MB despite the very diverse backgrounds of the investigated water samples from Lake Manzala^34^. In addition, statistical analysis (S10) showed that a weak effect of NH_4_^+^ and PO_4_^3−^ could be observed during winter, as concluded from weak correlations detected between the water quality parameters and the achieved *E*_*R*_%. This indicates the insignificant influence of background on the adsorption process. S11 shows a practical demonstration of MB-spiked waters before and after treatment as an evidence of the validity of this removal method.

## Conclusion

The seagrass dead leaves were effectively activated via a simple and rapid acetic acid treatment which led to a release of the acidic OH groups in addition to partial hydrolysis of the ester groups. SG_a_ lost more than 75% of its ash content compared with SG_p_ which is attributed to the release of metal and metalloid ions. Also SG_a_ acquired almost twice the surface area of SG_p_ with a remarkable increase in the acidity.

A maximum removal efficiency of >95% was achieved for both MB and Pb^2+^ on SG_a_ which was higher than SG_p_ by 3.6%. The optimum conditions for MB and Pb^2+^ adsorption onto SG_a_ are pH ranges of 2–12 and ≥6, and a dosage 3.0 and 0.5 g L^−1^, respectively at a shaking time of 30 min, which are suitable for a wide range of wastewaters.

The equilibrium data for MB and Pb^2+^ adsorption on SG_a_ were in agreement with Langmuir, Freundlich and DRK isotherm models, except for Langmuir model fitting in case of MB. However, the DRK and Freundlich isotherm models respectively, best described the adsorption processes of MB and Pb^2+^.

The adsorption onto SG_a_ was suggested to be an endothermic process. A multilayer physicochemical adsorption of MB onto SG_a_ is assumed for the adsorption of MB onto SG_a_ whereas Pb^2+^ is chemically adsorbed. The kinetics of the adsorption process were suggested to follow the pseudo-2^nd^-order kinetic model that is limited by the rate of adsorbate diffusion in the pores of SG_a_.

The removal process was successfully applied in MB-spiked brackish wastewater from Lake Manzala. Hence, the acid-activated seagrass powder can be effectively used as a cost-free adsorbent for the removal of MB as a cationic dye and Pb^2+^ from polluted brackish water with high capacities up to 2681.9 and 631.13 mg g^−1^, respectively.

## Supplementary information

Supplementary information
