# Supplementary material for: Adsorption of Methylene Blue and Pb2+ by using acid-activated Posidonia oceanica waste
Source: Sci Rep. 2019 Mar 4;9:3356. doi: 10.1038/s41598-019-39945-1 (PMC6399213; doi:10.1038/s41598-019-39945-1)
Supplement: Supplementary file 1 — Supplementary information [file 41598_2019_39945_MOESM1_ESM.docx]

Adsorption of Methylene Blue and Pb^2+^ by using acid-activated *Posidonia oceanica* waste

Randa R. Elmorsi,^a^ Shaimaa T. El-Wakeel,^b^ Waleed A. Shehab El-Dein,^c^ Hesham R. Lotfy,^d*^ Wafaa E. Rashwan,^e^ Mohammed Nagah,^f^ Seham A. Shaaban,^g^ Sohair A. Sayed Ahmed,^e^ Iman Y. El-Sherif,^b^ Khaled S. Abou-El-Sherbini^h,*^

1. Marine Chemistry Lab., National Institute of Oceanography and Fisheries, Suez branch, Adabiyah-Suez road, Attaqa district, Suez, Egypt. Email: rrelmorsi@hotmail.com.
2. Department of Water Research, National Research Centre, 33 El Bohouth st. (former Eltahrir st.), P.O. 12622, Dokki, Giza, Egypt. Email: shaimaa_tw@yahoo.com, iman_57us@hotmail.com.
3. Department of Mathematics & Engineering Physics, Faculty of Engineering, Mansoura University, El-Mansoura, Egypt. Email: waleedazmy1967@yahoo.com.
4. Basic Sciences Department, Faculty of Engineering, Delta University, Coastal High Way, Gamasa, Al-Dakahlia, Egypt. Email: heshamrabielotfy@yahoo.com.
5. Department of Physical Chemistry, National Research Centre, 33 El Bohouth st. (former Eltahrir st.), P.O. 12622, Dokki, Giza, Egypt. Email: wafaae.rashwan@yahoo.com, sohairabdelaziz@yahoo.com.
6. Department of Microbial Chemistry, National Research Centre, 33 El Bohouth st. (former Eltahrir st.), P.O. 12622, Dokki, Giza, Egypt. Email: moh_nagah_nrc@yahoo.com.
7. Department of Catalysis, Petroleum Refining Division, Egyptian Petroleum Research Institute, Egypt. Email: sehamshaban@yahoo.com.
8. Department of Inorganic Chemistry, National Research Centre, 33 El Bohouth st. (former Eltahrir st.), P.O. 12622, Dokki, Giza, Egypt. Email: kh_sherbini@yahoo.com. Fax: 0020233370931

**S1:**


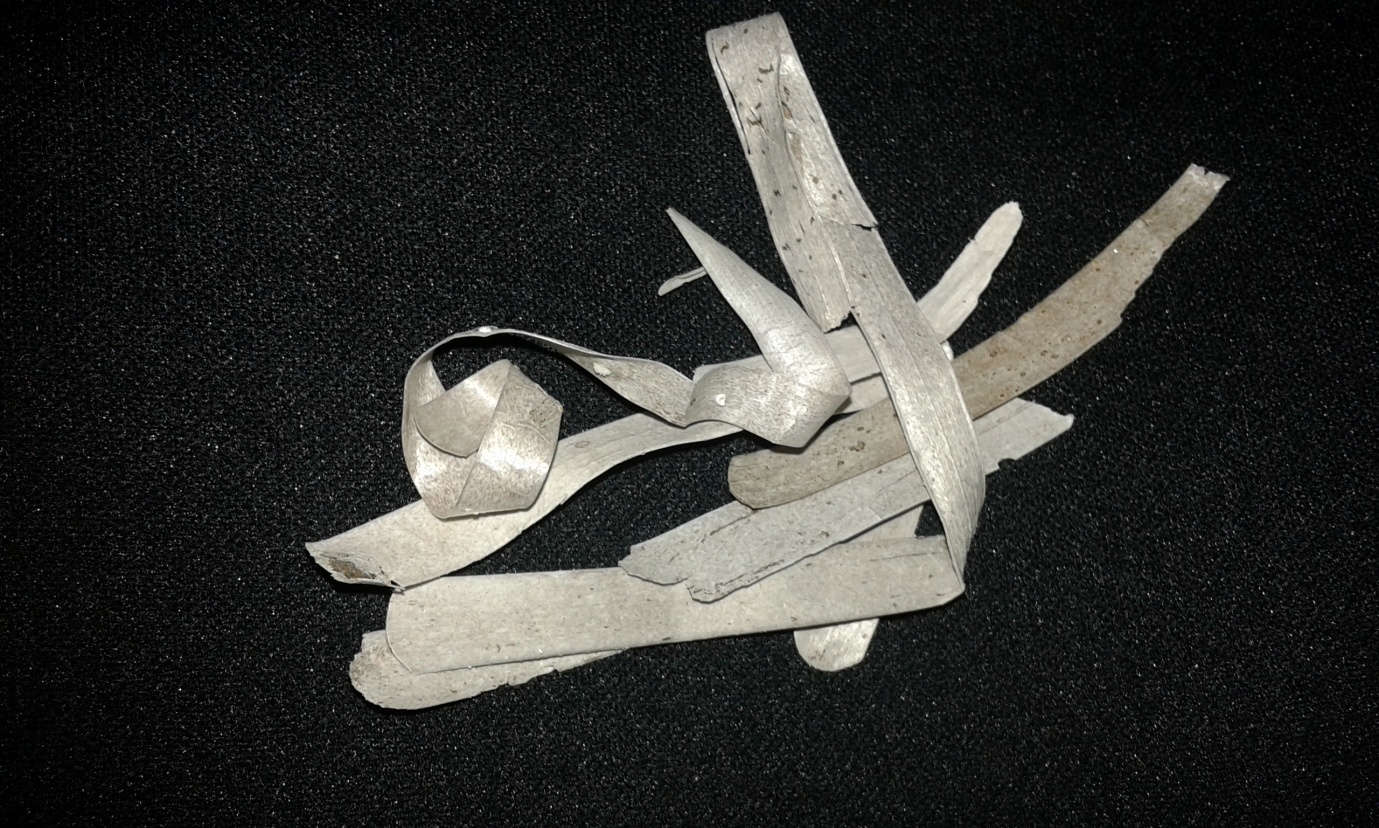


**S1.** SG dead leaves

**S2. Point of zero charge:** The point of zero charge (pH_PZC_) of the adsorbents was determined by adjusting the initial pH of 0.1 M NaCl solutions to a range of 2-12 using 0.01 M HCl or NaOH. Then, 25 mg of the different adsorbents was added to each solution. The suspensions were shaken in closed bottles for 24 h, and the final pH of the solutions was measured. The pH_PZC_ was then obtained from the intersection of the actual and theoretical graphs obtained by plotting the initial and final pH values.

**S3: Chemical composition of raw material.** Prior to analysis of the organic composition, the SG was washed several times in hot DW (80 °C) to remove free non-structural sugars ^1,2^, and then skimmed and dried in an oven at 45 ᵒC.

The lignin content was determined by sodium chlorite/acetic acid method^2^ and the hemicellulose content was determined by alkali extraction.^3^ Determination of the total cellulose was performed on the dried residue from the hemicellulose determination step using the monoethanolamine method.^4^ Moisture determination was performed on 5 g of the sample according to the method described in the literature.^5^ The ash content was determined by ignition of the sample in a muffle furnace at a temperature of 575 +/- 25 °C for 2 h.^5^

**S4:**

**S5:**

**S5:** Nitrogen adsorption (black square) – desorption (blue circles) isotherms of SG_p_ (up), SG_a_ (down) adsorbents

**S6:** The experimental adsorption time data were applied to two simplified kinetic models. The pseudo-1^st^-order model is described in the following equation:^6^

$ln\left( q_{e}-q_{t} \right)=lnq_{e}-K_{1}t$ (4)

The pseudo-2^nd^ order model is described in the following equation ^7^:

$\frac{t}{q_{t}}=\frac{1}{{{K_{2}q}_{e}}^{2}}+\frac{1}{q_{e}}t$ (5)

where k_1_ and k_2_ are the pseudo-1^st^- order (min^-1^) and pseudo-2^nd^-order (g mg^-1^ min^-1^) adsorption rate constants, respectively.

**S7:** The linear form of Langmuir equation (Eq. 1) is represented as:^8^

$\frac{\text{C}_{\text{e}}}{\text{q}_{\text{e}}}\text{=}\frac{\text{1}}{\text{bq}_{\text{max}}}\text{+}\frac{\text{C}_{\text{e}}}{\text{q}_{\text{max}}}$ (1)

where q_e_ is the amount of adsorbate per unit mass of adsorbent (mg g^-1^), C_e_ is the equilibrium concentration of metal ions in solution (mg L^-1^), q_max_ is the monolayer adsorption capacity (mg g^-1^) and b is a constant related to the free energy of adsorption. The essential characteristics and feasibility of the Langmuir isotherm can be described by a dimensionless equilibrium factor, *R_L_*, that is given by Eq. (2) as follows:

$R_{L}=\frac{1}{1+bC_{i}}$ (2)

The linear form of the Freundlich equation (Eq.7) ^9^ is represented as follows:

$\ln q_{e}=\ln K_{F}+\frac{1}{n}\ln C_{e}$ (3)

where K_f_ (L^3^ g^-1^) is the Freundlich constant, which indicates the relative adsorption capacity of the adsorbent (mg/g),and (*1/n*) is an empirical parameter that indicates the intensity of adsorption.

The DRK isotherm ^10,11^ is expressed as follows:

$q_{e}=q_{max}e^{\frac{\varepsilon^{2}}{-2E^{2}}}$ (4)

Where $\varepsilon$ is the adsorption potential, which$=RTln\left( \frac{1}{1+C_{e}} \right)$, R is the gas constant (8.314 J mol^-1^ K^-1^), T is the temperature (K) and E is the mean energy of adsorption, which provides information about the physical and chemical features of adsorption. The linear form of the DRK isotherm equation is as follows:

$lnq_{e}=lnq_{max}-\frac{\varepsilon^{2}}{2E^{2}}$ (5)

**S8**. Comparison of adsorption parameters obtained for the present system in comparison with the reported values

| Adsorbent | pH_pzc_ | pH | q_max,_ mmol g^-1^ | Dosage, g L^-1^ | | t, min | | AIM | | KM | | Ref |
| --- | --- | --- | --- | --- | --- | --- | --- | --- | --- | --- | --- | --- |
| MB | | | | | | | | | | | | |
| Lignocellulosic/neem oil-phenolic resin | 6.8 | 2-8 | 6.253 | 10 | | 5 | | L | | 2^nd^ | | ^12^ |
| Pycnoporus sp./oiltea shell | - | 3 | 0.227 | - | | 180 | | L | | 2^nd^ | | ^13^ |
| Trametes versicolor/oiltea shell | - | 8 | 0.268 | - | | 180 | | L | | 2^nd^ | | ^13^ |
| H_2_Ti_3_O_7_/γ-Fe_2_O_3_ | 3.0 | 10 | 0.238 | - | | 5.0 | | L, DRK | | 2^nd^ | | ^14^ |
| citrus limetta peel | 8.0 | >8.0 | 0.711 | 2.0 | | 30 | | L | | 2^nd^ | | ^15^ |
| SG activated carbon | - | 3-10 | 0.893 | - | | 60 | | L | | 2^nd^ | | ^16^ |
| SG | - | - | 0.388-1.506 | - | | - | | T | | - | | ^17^ |
| SG_a_ | 6.6 | 2-12 | 6.435 | 3.0 | | 30 | | F, DRK | | 2^nd^ | | This work |
| Pb^2+^ | | | | | | | | | | | | |
| *Landoltia punctuate* | 7.1 | 4.6 | 1.2 | | 4.0 | | 100 | | L | | 2^nd^ | ^18^ |
| *Spirodela polyrhiza* | - | 4.6 | 0.97 | | 10.0 | | 30 | | L | | 2^nd^ | ^18^ |
| Activated carbons | 5.7, 6.2 | - | 0.24, 0.222 | | - | | 30 | | L | | 2^nd^ | ^19^ |
| Xanthate/chitosan/poly(vinyl alcohol) | - | 6.88 | 0.289 | | - | | - | | L, F | | 1^st^, 2^nd^ | ^20^ |
| lignin grafted carbon nanotubes | 4.4 | 7.0 | 0.739 | | 0.5 | | 50 | | HL | | 2^nd^ | ^21^ |
| SG | - | 4.0-5.0 | 0.269 | | - | | 20 | | J | | 1^st^, 2^nd^ | ^22^ |
| Succinic anhydride-modified SG | - | 4.0-5.0 | 1.052 | | - | | 20 | | J | | 1^st^, 2^nd^ | ^22^ |
| SG | - | 2.0-3.0 | 0.240 | | - | | 20 | | L | | 2^nd^ | ^23^ |
| SG_a_ | 6.6 | ≥ 6 | 3.046 | | 0.5 | | 30 | | L, F, DRK | | 2^nd^ | This work |

The symbols t, AIM, KM, ref, L, F, E, HL, J, 1^st^ , 2^nd^ and T denote equilibration time, adsorption isotherm model, kinetic model, reference, Langmuir, Freundlich, Elovich, Hyper-Langmuir, Jossen, pseudo 1^st^ order, pseudo 2^nd^ order and Thomas (column technique) models, respectively.

**S9: The removal efficiency of MB from Manzala Lake water samples using SG_a_**

| Location | Removal Efficiency (%) | |
| --- | --- | --- |
|  | Winter | Summer |
| 1 | 95.6 | 95.6 |
| 2 | 99.1 | 98.8 |
| 3 | 98.2 | 98.6 |
| 4 | 91.8 | 93.9 |
| 5 | 97.3 | 94.6 |
| 6 | 99.9 | 98.5 |
| 7 | 98.7 | 99.9 |
| 8 | 99.8 | 99.9 |
| 9 | 95.6 | 91.5 |
| 10 | 98.5 | 99.0 |
| 11 | 98.8 | 99.1 |
| 12 | 98.7 | 99.8 |

Mass of SG_a_ 100 mg, volume 20 mL, initial concentration of MB 40 mg/L.

**S10: Pearson correlations of the removal efficiencies of MB onto SG_a_ from the investigated Manzala Lake water samples with their quality parameters**

| Parameter | | Winter | Summer |
| --- | --- | --- | --- |
| pH | Pearson Correlation | 0.399 | -0.378 |
|  | Sig. (2-tailed) | 0.198 | 0.226 |
|  | N | 12 | 12 |
| EC | Pearson Correlation | 0.308 | 0.055 |
|  | Sig. (2-tailed) | 0.331 | 0.864 |
|  | N | 12 | 12 |
| TDS | Pearson Correlation | 0.296 | 0.045 |
|  | Sig. (2-tailed) | 0.351 | 0.890 |
|  | N | 12 | 12 |
| Salinity | Pearson Correlation | 0.297 | 0.056 |
|  | Sig. (2-tailed) | 0.349 | 0.863 |
|  | N | 12 | 12 |
| DO | Pearson Correlation | 0.447 | 0.398 |
|  | Sig. (2-tailed) | 0.145 | 0.200 |
|  | N | 12 | 12 |
| BOD | Pearson Correlation | -0.513 | -0.500 |
|  | Sig. (2-tailed) | 0.088 | 0.098 |
|  | N | 12 | 12 |
| MO_4_^-^ index | Pearson Correlation | -0.466 | -0.284 |
|  | Sig. (2-tailed) | 0.127 | 0.371 |
|  | N | 12 | 12 |
| Chloride | Pearson Correlation | 0.277 | 0.058 |
|  | Sig. (2-tailed) | 0.384 | 0.857 |
|  | N | 12 | 12 |
| Alkalinity | Pearson Correlation | -0.201 | 0.556 |
|  | Sig. (2-tailed) | 0.532 | 0.061 |
|  | N | 12 | 12 |
| NH_4_^+^ | Pearson Correlation | -0.657(*) | -0.497 |
|  | Sig. (2-tailed) | 0.020 | 0.100 |
|  | N | 12 | 12 |
| NO_2_^-^ | Pearson Correlation | 0.215 | -0.123 |
|  | Sig. (2-tailed) | 0.502 | 0.703 |
|  | N | 12 | 12 |
| NO_3_^-^ | Pearson Correlation | 0.153 | -0.168 |
|  | Sig. (2-tailed) | 0.635 | 0.602 |

**S10: Continued.**

| Parameter | | Winter | Summer |
| --- | --- | --- | --- |
|  | N | 12 | 12 |
| TIN | Pearson Correlation | -0.504 | -0.492 |
|  | Sig. (2-tailed) | 0.095 | 0.104 |
|  | N | 12 | 12 |
| PO_4_^3-^ | Pearson Correlation | -0.690(*) | -0.406 |
|  | Sig. (2-tailed) | 0.013 | 0.190 |
|  | N | 12 | 12 |
| PAHs | Pearson Correlation | .224 | 0.068 |
|  | Sig. (2-tailed) | 0.485 | 0.834 |
|  | N | 12 | 12 |

** Correlation is significant at the 0.01 level (2-tailed).

* Correlation is significant at the 0.05 level (2-tailed).

**S11**


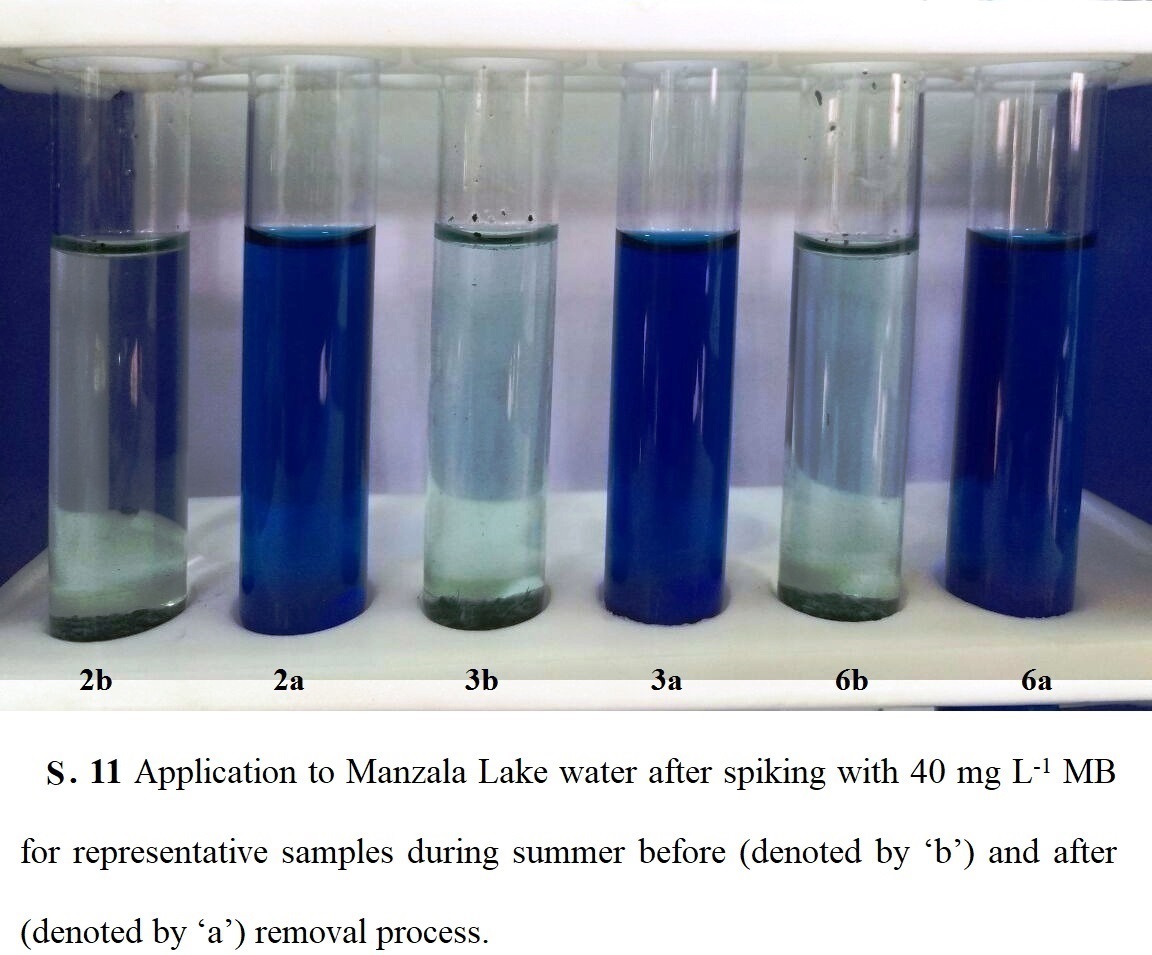


Reference

1. Garlock, R. J. *et al.* Comparative material balances around pretreatment technologies for the conversion of switchgrass to soluble sugars. *Bioresour. Technol.* **102,** 11063–11071 (2011).

2. Shi, J. *et al.* Application of cellulase and hemicellulase to pure xylan, pure cellulose, and switchgrass solids from leading pretreatments. *Bioresour. Technol.* **102,** 11080–11088 (2011).

3. Carr, R. L. C. M. E. & Bagby, M. O. Hemicellulose Isolation from Annual Plants *. *Biotechnol. Bioeng.* **17,** (1987).

4. L. Nelson and J. A. Leming. Evaluation of monoethanolamine method of cellulose determination for agricultural residues. *Tappi J.* **40,** 846–850 (1957).

5. Sluiter, a *et al.* Determination of total solids in biomass and total dissolved solids in liquid process samples. *Natl. Renew. Energy Lab.* 9 (2008). doi:NREL/TP-510-42621

6. Marques, S. C. R., Marcuzzo, J. M., Baldan, M. R., Mestre, A. S. & Carvalho, A. P. Pharmaceuticals removal by activated carbons: Role of morphology on cyclic thermal regeneration. *Chem. Eng. J.* **321,** 233–244 (2017).

7. Ho, Y. S. & McKay, G. Pseudo-second order model for sorption processes. *Process Biochem.* **34,** 451–465 (1999).

8. Langmuir, I. The adsorption of gases on plane surfaces of glass, mica and platinum. *J. Am. Chem. Soc.* (1918). doi:10.1021/ja02242a004

9. Freundlich, H. M. . Over the adsorption in solution. *J. Phys. Chem* (1906). doi:10.3390/ijerph9030970

10. Cortes, J. & Araya, P. The Dubinin-Radushkevich-Kaganer Equation. *J . Chem. SOC. Furaday Trans. I* (1986). doi:10.1039/F19868202473

11. Dubinin, M.M. and Radushkevich, L. V. The Equation of the Characteristic Curve of Activated Charcoal. *Proc. Acad. Sci. Phys. Chem. Sect.* (1947). doi:10.4236/ojs.2014.41001

12. Manna, S., Roy, D., Saha, P., Gopakumar, D. & Thomas, S. Rapid methylene blue adsorption using modified lignocellulosic materials. *Process Saf. Environ. Prot.* **107,** 346–356 (2017).

13. Liu, J., Li, E., You, X., Hu, C. & Huang, Q. Adsorption of methylene blue on an agro-waste oiltea shell with and without fungal treatment. *Sci. Rep.* **6,** (2016).

14. Jose, M. *et al.* Ion-exchange bonded H 2 Ti 3 O 7 nanosheets-based magnetic nanocomposite for dye removal via adsorption and its regeneration via synergistic activation of persulfate. *RSC Adv.* **6,** 80133–80144 (2016).

15. Shakoor, S. & Nasar, A. Removal of methylene blue dye from artificially contaminated water using citrus limetta peel waste as a very low cost adsorbent. *J. Taiwan Inst. Chem. Eng.* **66,** 154–163 (2016).

16. Dural, M. U., Cavas, L., Papageorgiou, S. K. & Katsaros, F. K. Methylene blue adsorption on activated carbon prepared from Posidonia oceanica (L.) dead leaves: Kinetics and equilibrium studies. *Chem. Eng. J.* **168,** 77–85 (2011).

17. Cavas, L., Karabay, Z., Alyuruk, H., Doĝan, H. & Demir, G. K. Thomas and artificial neural network models for the fixed-bed adsorption of methylene blue by a beach waste Posidonia oceanica (L.) dead leaves. *Chem. Eng. J.* **171,** 557–562 (2011).

18. Tang, J., Li, Y., Wang, X. & Daroch, M. Effective adsorption of aqueous Pb2+by dried biomass of Landoltia punctata and Spirodela polyrhiza. *J. Clean. Prod.* **145,** 25–34 (2017).

19. Nieto-Márquez, A., Pinedo-Flores, A., Picasso, G., Atanes, E. & Sun Kou, R. Selective adsorption of Pb2+, Cr3+ and Cd2+ mixtures on activated carbons prepared from waste tires. *J. Environ. Chem. Eng.* **5,** 1060–1067 (2017).

20. Lv, L., Chen, N., Feng, C., Zhang, J. & Li, M. Heavy metal ions removal from aqueous solution by xanthate-modified cross-linked magnetic chitosan/poly(vinyl alcohol) particles. *RSC Adv.* **7,** 27992–28000 (2017).

21. Li, Z., Chen, J. & Ge, Y. Removal of lead ion and oil droplet from aqueous solution by lignin-grafted carbon nanotubes. *Chem. Eng. J.* **308,** 809–817 (2017).

22. Chadlia, A., Mohamed, K., Najah, L. & Farouk, M. M. Preparation and characterization of new succinic anhydride grafted Posidonia for the removal of organic and inorganic pollutants. *J. Hazard. Mater.* **172,** 1579–1590 (2009).

23. Dridi-Dhaouadi, S., Douissa-Lazreg, N. Ben & M’Henni, M. F. Removal of lead and Yellow 44 acid dye in single and binary component systems by raw Posidonia oceanica and the cellulose extracted from the raw biomass. *Environ. Technol.* **32,** 325–340 (2011).
